# Silicious Cement

**Published:** 1909-06-15

**Authors:** W. V-B. Ames

**Affiliations:** Chicago


### SILICIOUS CEMENT.

BY W. V-B. AMES, D.D.S., CHICAGO.

Read before the Michigan First District Dental Society, May 8, 1909.

I am compelled to come to you to-night with a sort of apology, inasmuch as the discussion requested of me happens to be of such a live and vital character at this time that it has become more or less necessary for me, on two or three occasions within the year to take part in discussions on this particular subject, so that in the April issue of the *Dental Cosmos* appears considerable matter which I intended to bring to you.

Last November I was asked to take part in discussing this subject before the New York Otological Society, and if I had anything worth saying down there I had to say much of what I bring you to-night, except I can say that the real vital part and valuable part of what I bring to you to-night I was not able to give at that time and have not been able to give at any time previous it being the result of some experiments I have been carrying on to ascertain the reasons for some of the shortcomings and vital defects of the so-called Silica cements. Of these shortcomings we shall have something to say as we go along.

---

Probably some of you recall attempts at making a paste to take the place of the cement liquid. One you may remember the Samil Hussy paste acid, which passed into oblivion in a short time, the same as the Fletcher paste and other similar attempts. It is quite possible that Fletcher's materials were faulty because of the attempt to use this paste instead of the acid solution.

For fear that there is a rather general impression that the so-called silicate cements differ wholly and totally from the phosphate cements, I want to begin with the statement that the silicious cements of to-day are all oxyphosphates just as much as are the zinc cements with which you are familiar, the silicious variety being oxyphosphates of calcium modified by silica, and alumina in all cases, and other substances as the manufacturer is moved.

It will probably be of some interest to review the known attempts at the production of this kind of a phosphate. An early record of a tooth plugging and pulp capping material is the patent of Wm. H. Rollins, of Boston, Mass., application on which was made March 28th, 1879. This was not an oxyphosphate but more of a hydraulic cement of the Portland variety, in which the setting depended on treating a mixture of calcium oxid and silicates with water, the water for the Rollins material containing some gelatinous silica. I mention this because this material has been cited as an early example of silicious phosphate.

The earliest record of a *real oxyphosphate* of the silicious calcium variety that I have been able to obtain, is the patent of Thos. Fletcher, of Warrington, England, application on which was filed July 29, 1879. This specification calls for an acid solution of phosphate of tin which will react with a compound prepared by fusing together lime silica and alumina. I am of the belief that the Fletcher preparation failed of marked success partly because of the instability of the acid solution of phosphate of tin. Another Fletcher patent of the same year calls for an acid paste containing aluminum phosphate. The history of such *acid pastes* for dental cements indicates negative value.

Some ten years later an oxyphosphate of the silicious calcium variety began to be shown by Dr. McGeorge, of Corning, N. Y., and was later put upon the market by The S. S. White Co., as "Dentos." A few years after this "Arcite" appeared through Lee S. Smith & Son, this also being of the silicious calcium variety. With these two efforts, the principal fault appeared to be the inevitable shrinkage taking place in a mass during the setting process, and in the early material produced by Steenbock, of Berlin, under the name Ascher enamel, this fault was evident to a fatal degree. This Steenbock material is a silicious calcium oxyphosphate, said to be radically improved over any other possible material, because of a beryllium silicate ingredient. Since this many similar preparations have appeared including Astral, Howerdid, Smaltid, Hoffmans Enamel, Lorenz Silikat, Schoenbecks Silicate and some others.

On February 12, 1906, an application for patent was filed in the United States by Josef Rawitzer, of Charlottenberg, Germany, and patent granted same year. In this specification, the material being that known as Astral, Rawitzer says that a superior cement with reference to brittleness and shrinkage can be made by adding aluminum silicate prepared in a wet way to molten silicate of lime and aluminum, and it is on this preparation of aluminum silicate in a wet way, that the U. S. patent office granted letters patent, which seems a joke to the layman, as the wet way had no doubt been the natural way of preparing aluminum silicate in the making of previous similar preparations.

In the specifications for the patents of Steenbock issued in 1904, some extravagant claims are made for beryllium silicate as the distinguishing ingredient of his cement. and in advertising matter appearing from time to time, it is claimed that because of this ingredient, his cement "stands as the only absolutely perfect filling material." It is not for me to pass on the value of the different silicious calcium oxyphosphates, but I feel safe in giving some working suggestions, which I believe can be followed to advantage with all materials of this cless.

While I know that too extravagant statements have been made by manufacturers, which do not enhance the dignity of even a commercial pursuit, I am of the opinion that, while the perfect product is far from a realty, such advances have been made since Fletcher's first attempt, that the prospects are encouraging. The advance toward reliable materials has been particularly rapid during the past two years or since Steenbock brought out his improved Ascher Enamel which I consider is a marked approach to the ideal material wished for. The makers have brought out their improved, and their comparatively and superlatively improved editions of their original attempts. With the variety of shortcomings, among which we may mention lack of adhesion, shrinkage, and brittleness (causing chipping at edges) and a tendency with some materials to discolor badly in the mouth, I predict that this generation will not see all of the improvements in this particular field. I can predict this with plenty of courage of conviction knowing that the ramifications of possible formulae are multitudinous. The alloy question it has been said, is a Sunday picnic compared with that of oxyphosphate of zinc and I can easily say that the same comparison might be made between the zinc phosphate situation and that of producing a high grade silicious cement.

The latest patent on anything of this kind granted to W. V-B, Ames, a fluid silicate and phosphate.

It happens that I am able to produce a material which shows some adhesion, is free of shrinkage, so that in its use undercuts are not called for, has a fair degree of translucency, does not discolor in even an unclean mouth, will withstand severe abrasion and acid and alkaline tests and has some desirable working qualities, and yet I probably have only scratched the surface of the possible field, and would be very loathe to say that any one to-day has produced the perfect material.

Cavity preparation, where the use of cement is justified, should be, I believe, a very radical departure from the extension for preventive method, which has become gospel with the successful gold workers of to-day. For gold fillings and for gold and porcelain inlays, enamel margins have properly been cut back very freely. For best results with a Silicious calcium oxyphosphate, I am ready to lay down a rule that any bit of enamel that does not render impossible the proper cleansing of the cavity and that does not render the cavity margin too grotesque, should be retained. With an equipment of excavators such as Darby Perry 1-2-3-9-10, the Gillett 4-5-6-7, Battle Ax 1-2-3-4-5-6, and others calculated to remove caries from inaccessible locations, proper cavity preparations is fairly simple after separation for approximal cavities, with the *retention* of enamel, which would be cut away for operations with some other materials. In making these statements we have approximal cavities of incisors mostly in mind.

In treating cavities in bicuspids or molars, reaching to the occlusal surface, if enamel can be retained which will give cavity edges removed from direct occlusion, and give an acute instead of an obtuse edge of enamel against which to finish the cement, the prospects are much lessened of having an objectionable angular annoyance presented to the tongue from *slight* wear of the cement.

In connection with the making of silicious cement fillings, I want to call attention to the value of oxychlorid of zinc as a cavity lining and as a pulp protection. Silicious cements are excellent conductors of thermal changes, so that a proper non-conductor will be often called for, and very often an intermediate layer having germicidal properties will be indicated. A silicious cement cannot be counted on for even the sterilizing properties of oxyphosphate of zinc because of its inert ingredients and because the compound is not acted upon by the reagents present in the mouth. I think that oxychlorid of zinc fulfills the requirements more thoroughly and with fewer objections than any material available. It can be prepared to set quickly, especially with the application of heat. It is likely to be irritating temporarily, but can be used, I believe, over any pulp which is fit to be retained, with in rare cases a coating of some bland material directly over an almost direct exposure. I wish to especially emphasize the fact that silicious cements are prime conductors of thermal changes, if they have the desired texture.

Oxychloride of zinc shoufd be mixed to a hard consistance to secure prompt setting.

Silicious cements should be manipulated with non-corrosive instruments. An agate spatula, I consider, the only proper instrument for the mixing process. Bone or ivory are so rapidly abraded, that for that reason their use is constraindicated, finely comminuted bone not being a valuable addition to a cement mass. A slab with a slightly etched surface, I have found an advantage, as with a perfectly smooth slab, the mass as it acquires some stiffness, is apt to slide to an objectionable extent.

Some of the silicious cements are so susceptible to a hastening of setting from a rise of temperature, that a decided advantage obtains from working upon a chilled slab. A cement which would be objectionably quick setting when mixed on a slab of room temperature will admit of the incorporation of more powder and be rather slow of setting when mixed on a chilled slab.

I wish at this point to repeat what may be found in the May *Cosmos* regarding the mixing of these materials. There has been offered much faulty instruction as to manipulation. I do not believe that a rational process for mixing and using has yet been described, and I am sure that many fillings have been ruined by following the instructions calling for the manipulation and disturbance of the cement mass during the setting process, this disturbance being carried out by means of vaseline coated burnishers. This process, I think, has been abandoned to a considerable extent because a great many fillings were ruined by the process. By stopping to think of what would be the result of so interfering with the crystallization process, it is easy to see that it has been faulty instruction in the face of our knowledge of such processes in general. We do not know too much about crystallization, but we do know that the process of crystallization should not be disturbed after it begins to take place. I am sure that this material should be mixed according to the common knowledge possessed in mixing other materials thoroughly to a point from which it might be expected you would get the maximum of strength and then after it has been placed accurately in the cavity and given proper form, by the application of heat, and without further disturbing, the setting should be hastened and caused to take place in as short a time as possible consistent with the comfort of the patient. The mass, after it has hardened, should be finished as much as may be desired and with considerable care. There is usually some finishing necessary. There will be obtained by this process a cement mass of an integrity superior to that secured by following any set of instructions before offered in connection with any marketed product, and without securing the usual shortcomings. Where 15, 20, or 30 minutes is advised in the insertion and treatment of these fillings, by the process of burnishing with vaseline burnishers, I think with any materials $\frac{1}{3}$ to $\frac{1}{6}$ of this time will be sufficient for proper crystallization if heat be applied by any advantageous scheme, such as projecting by hot air, or the application of hot paraffine on the surface. This heat may be applied directly to the cement mass, in the angles of large restoration of bicuspids and molars, or any other cases, the mass being condensed beneath celluloid. The celluloid should be removed as soon as the cement will permit without distortion of the filling, but the heat may be applied while the celluloid strips is still in place. I think both vaseline and cocoa-butter are abominations used in connection with these materials, and that the mass of cement burnished with vaseline burnishers locks better on dismissal after that treatment than it ever does again after the grease dissappears from the surface. A substitute for the vaseline is paraffine, such as I often choose in using heat to harden cement. A strip of sand paper, or emery strip drawn across a lump of paraffine puts it in ideal condition, or rotating a disc on a lump of paraffine does the same work. Paraffine is primarily used as a means of transmitting heat to the mass to hasten its setting. In observing the effect upon pellets, not used for fillings, of this molten paraffine, it seemed evident that there was some chemical action between paraffine in the molten condition and free phosphoric acid. Recently I made the experiment of heating together paraffine and phosphoric acid and there is no doubt that the temperature required for melting the paraffine produced a slight chemical combination. I think that the free phosphoric acid on the surface of the cement mass can be neutralized by the paraffine at that temperature, so that I do not hesitate in saying that I firmly believe that in addition to hastening the setting there is a neutralization by the heated paraffine of the slight amount of free phosphoric acid resulting from what may be present with a proper mix.

I think that the cement can usually be inserted into the cavity with steel instruments without serious damage, as with cement of proper plasticity there would not be an abrasion of the metal although I would consider an absolute result worth the expenditure necessary to procure non-corrosive points of proper shapes. For the filling of small approximal cavities, some delicate instruments are called for which cannot be formed from agate or even bone or tortoise shell. Such may be formed from iridio platinum and set into steel shanks. A practical make shift is the coating of steel instruments with celluloid or cellulose, by dipping the instrument into a solution of a good collodion and allowing the pelicle to harden before use. By cleaning the instruments while the cement is quite fresh, a good coating of a good liquid court plaster such as that called "New Skin" might be equal to many operations. For burnishing surfaces for moulding to form, or for finish, agate is ideal, but in a majority of cases with approximal cavities of anterior teeth representing the majority, I would say the cement may be finally manipulated by steel burnishers with thin celluloid interposed between burnisher and cement. These strips are furnished and are no doubt familiar. Ash & Sons, while not successful with Astral, did furnish good appliances for its use. Vaseline, I think an abomination as an adjunct to the making of silicious cement fillings. The only excuse for vaseline known to me is that by its use a filling may be burnished to present a better appearance than it ever will after the grease dissappear. Paraffine I find much more satisfactory for the lubrication of discs and strips than vaseline, cocoa-butter or any such material previously recommended. It may be employed by drawing the strip or revolving the disc upon a lump of paraffine. I still believe the mere end thrust of a steel instrument will not cause much harm in the way of discoloration.

The setting of some of these cements can be decidedly accelerated by a rise of temperature, without detracting from the integrity. After a cavity is properly full of properly condensed cement, heat may be applied as hot air, or hot paraffine, with the result of very quick setting. If the cement has been moulded beneath thin celluloid, the heat may be applied upon the celluloid, the strip being easily removed in a short time (probably about one minute) without distorting the cement surface, and without the need of vaseline coating although the merest film would not be objectionable. After finishing the hardened cement to the edges by cutting with knives, burs, strips or discs, a coating of paraffine is an advisable precaution against saliva for a few hours. As finished after this plan, I consider that the mass has crystallized to the extent of 90% or 99% the paraffine coating being advisable however as a protection during the final slight changes which probably progress for several hours. I feel safe in saying that with some cements where it is advised to wait thirty minutes or more for the hardening, a better result might be obtained by very much curtailing the time, as I have found the hot paraffine scheme an apparent advantage with materials with which a long wait and no heat was directed. A chilled mixing slab will quite generally be found advantageous from retarding the setting thus enabling the admixture of a greater amount of powder and more thorough spatulation. That disturbances of the cement after crystallization has progressed to a decided extent are wrong needs no argument. Instructions furnished with some silicious cements calling for this sort of procedure have been the cause of many inferior results.

Actual and decided undercuts are not needed as generally as some instructions seem to indicate. Some silicious cements as prepared during their early history did not need these undercuts because of their shrinking proclivities for which reason there was no sane reason for their use. As made at present these same cements will not shrink when properly handled, therefore only good cavity form is called for. since there is so little adhesion with some makes, at least parallel walls are needed, while if it is found that some preparations show some adhesion, then only fair cavity for will be needed as with any other adhesive cement. As to results, I have seen some sufficiently creditable to encourage one to proceed cautiously. I feel warranted in saying that the time has past for cutting back radically the enamel margins about a small approximal cavity for the insertion of a porcelain inlay, and for the management of labial and buccal cavities, these cements when free of the tendency to shrinkage and discoloration, are almost ideal.

When I prepared this paper I did not fully realize to what extent the trouble of discoloration was attributable. I knew that it did happen and with some makes of cement much more than with others. Until recently I had been inclined to think that the fault was with the dentists using it. I supposed that it probably came from the use of steel instruments to too great an extent, and possibly it was at the time that the Ascher enamel people were advocating the use of vaseline, for by and by they said, don't use vaseline, because that is the cause of the discoloration.

It has been my lot to hear a great many expressions about these materials, and no cases where fillings were the same as they were inserted. They were often matches to the teeth in which they were placed, and were satisfactory for a time, and then they began to change color and would need to be removed. It has been strongly urged in the use of these materials that the original surface be retained to as great an extent as possible, and it was for that reason that the early instructions called for the moulding of the material while it was setting, endeavoring to leave the cavity just full and needing no finishing, all of which did not convey any strong impression to me until in some recent tests I found that where I had previously tested cements dropping masses just as they had been moulded into a staining solution, the solution being composed of potassium sulphide, ammonia and sulphide, answering the same purpose, I was under the impression that the staining we hear of was not inherent in the material or not a necessary result, because those pellets would stay so clean in the solution, if the ordinary tentative discoloring of the mouth was discoloring these. I happened to find, in the course of time, with the least abrasion, such abrasive spots would immediately take on a stain, and that stain would rapidly permeate the mass, so that threw some light on this discoloring after they had been in place for a time. It indicated that as soon as they lost their original surface the stain penetrated much more rapidly. The surface is always a little different than the material beneath. One reason for it is that the free acid tends to come to the surface, and so there is an excess of crystallization. That would account for the surface difference, but I do not pretend to say here why in any particular cements that there is always a case hardening of a certain protection, but the fact remains that as soon as the surface is broken the stain rapidly increases. Now in addition to that I found in testing our various colors that there was a difference in the different colors and the extent of their discoloration. I found uniformly that the plain white enamel would stain less than the pigmented materials, and that some of the colors stained worse than the others. The Ascher material, for instance: the No. 6 is the plain white, and I have made a test say of the various colors, and you can see it as I pass these specimens about. There are five shades here, although I have tested more shades. Nos. 1 and 7 stained very badly, and became in spots and the thin edges became as soft as so much charcoal almost, from the effects of the sulphide solution. Nos. 1 and 7 staining badly, and Nos. 4 and 5 not so badly, Nos. 2 and 3 being intermediate. While it may be said that the strong solution of sulphide of potassium is not a fair test, I do not think it can reasonably be said that it is not a fair test. While it is extreme, it is, I verily believe, just an indication of what can take place in the mouth, and what does take place in the mouth to a lesser degree, and all this indicates to me that certain colors of these cements are ruined in the pigmenting process; they are rendered liable to the sulphuretting, and the pigmenting material aids the destruction to pass to the center of the mass. In testing different cements I found a difference in the form of the strain between, say, the Ascher material and some others not so well known. There is the Schonbeck, which is a material at present probably more used in Germany and England than the Ascher, and it is probably coming into use, because with it, while there is sometimes a tendency to stain and the same rule holds with the pigmenting the colors, the greys, yellows and blues stain more readily and more extensively than the plain white; it seems that it is more of a surface discoloration and that it is a sulphide which is soluble instead of being insoluble and tending to go deep into the mass. Now what will be the actual result of the test of time; all this remains to be seen, but in that respect it is promising that this stain does not go to the same depth, does not permeate the entire mass, and it seems that while there is a tendency with the colors to form sulphides, there seem to be soluble sulphide and the soluble sulphide, while it might color under some conditions, soluble sulphide would dissappear and you would not know it has been formed.

DR. DUMAS. How old are these fillings?

DR. AMES. Ordinarily I have these staining as soon as they seem to be sufficiently set. By means of heat I can cause any of these cements to be crystalized, to be submitted to water in solution in 10 or 15 minutes' time, and then they are left for 24 hours in the stain, and then, with the Ascher material, after taking them out of the stain and placing them in plain water the stain seems to progress. I believe that in one case it is an insoluble sulphide which tends to creep and continue, and when the other case, it is a soluble sulphide, which, in the mouth, would never be noticed because in this case it forms because it is in the sulphide potassium, a solution furnishing sulphide of hydrogen, and in the other case it is dissolved away as soon as it is formed. The fact remains that with the identical treatment with these cements, and all of them the unpigmented material stains less than the pigmented, and with one cement the stain seems to be soluble in water, in sulphide, in the other case it is insoluble, and then, of course, is a permanent stain.

### DISCUSSION.

Dr. L. P. Hall.—My acquaintance with Silicate cements began about a year and a half ago. My intention was to try Ascher's Artificial Enamel, but when I called for it at the supply house I was prevailed upon to try some Translux, being told it was just as good.

I had a nice old lady patient whose teeth protruded a good deal from recession of the gums and it did not seem best to use so much pressure as would be necessary in making inlays. I bought the Translux for that particular case.

I was so much pleased with the results that I ordered another shade and began using it in a few cases that I could see often and watch results. I soon found that the fillings changed in color, not in every case but in several cases a good deal. I then purchased some Ascher's and began practicing upon brother dentists. It was surprising how readily they consented to any kind of a plastic filling for themselves when I knew they always insisted upon either gold or porcelain for their patients. The most of these fillings have proved satisfactory.

I prepared a good many cavities in extracted teeth to see what would be possible in the way of reproducing stains and colors as we can with porcelain. By having an assistant mix one color while I prepared another some very satisfactory results were produced. I had as many failures in colors and in fillings as we all have in porcelain. In the mean-time I had had a chance to see the change in color in some Trans-lux fillings I had made for some of our students. These I replaced with Ascher's with considerable improvement in color and in manipulation.

I do not know of any preparation on the market which the makers have tried as hard and persistently to keep the users in touch with all the best and latest methods of using their preparation as have the Ascher people by means of their letters and bulletins.

The technique necessary to the making of a perfect filling of Ascher's Enamel is equal to that required for that of any material we have ever had. It is a very delicate process. It does not require a very elaborate lot of instruments but they must be just right, and the materials must be handled just so, and a considerable amount of speed must be developed to enable one to get the material into a cavity of any considerable size before the cement becomes too hard to allow of drawing it to the borders of the cavity. This must be accomplished before the setting has begun else, in drawing the cement to the border, the whole mass is likely to be disturbed and so leave some other border unfilled. Such a mistake might give rise to the belief that the product had shrunken after a few days.

The ideal way would be to put as nearly as possible the exact amount of cement into the cavity and then burnish under celluloid. It has not been my good fortune to get the exact amount very often, but, if one is careful not to use too coarse discs or strips in cutting down to nearly the desired surface the finer strips and discs will cut the filling down to a very nice surface.

I am thoroughly in accord with the essayist when he says, "Do not use vaseline on the instruments while inserting the material," but after it is in and while burnishing the filling it seems to me the vaseline serves a great purpose in preventing the rubber dam from being caught or cut and thus letting in moisture.

I have tried the suggestion of using paraffine on strips and discs and find it works very well. It is better than vaseline in one way as it fills up the spaces between the grit on the discs and strips and so does not allow them to cut quite so deeply into the surface of the filling. It is very important not to cut off too much of an overhanging border at a time, as the mass is so friable it is liable to fracture too far back, and so leave a fault on the surface. For the same reason it is never well to leave any portion overlapping a border, no matter how smoothly it seems to blend into the tooth surface. Sooner or later it will crack off and leave a rough border.

A short time after I began experimenting with Silicate cements I met a Dr. King who had practiced several years in Brazil. He told me that he had not put in a gold filling for the past three years. Before that he said their patients had been great sticklers for gold work but would have none of it now. It is only fair to add that up to that time they had done little if any porcelain work.

In June of last year I met Dr. Bodecker, of Berlin, and when I asked what they thought of Silicate cements over there he said they used them whenever there were four walls to a cavity. Such statements encouraged me to further experiments.

Unfortunately some of the anterior cavities do not have four walls and so, for experimental work, I prepared some of those cavities in extracted teeth. In these screws pins of german silver were inserted to help strengthen and retain the fillings as we do for restoring incisal angles with gold. When filled and polished they seemed so strong that I tried some threaded screws in patients teeth and it is gratifying to state that these incisal restorations are so far holding their own as well as porcelain would in the same cavities.

By the use of a matrix I have used Ascher's material in filling compound cavities in molars and bicuspids. The matrix, if metal, should be just touched with vaseline and then wiped almost dry so that no particle of vaseline will adhere to the tooth. If a celluloid matrix is used it will leave the cement as soon as the latter is set without the aid of vaseline.

In any cavity provision must be made to protect against thermal changes, but in molars and bicuspids the intermediate filling must not be so thick as to leave room for *only a thin layer* of the Silicate cement. Occlusal fillings should be as thick in proportion as possible. Any thin portion extending into fissures or over occlusal borders is liable to fracture. Also occlusal surfaces should be dressed down as nearly as possible to the normal occlusion before removing the dam. There is no give to one of these fillings as there is in either gold or amalgam fillings.

Last summer I did some Ascher's fillings for a young lady who came in for more work the other day and I found two compound fillings cracked. It took me a full hour to remove the hardened cement from one of them. I had not had any cracking of these fillings before and I am of the opinion it was due to the fact that the patient did not appreciate that the fillings were too high and so did not inform me correctly before leaving the chair.

In cavity preparation the essayist urges very strongly his rule to retain every bit of enamel wall. For various reasons that would be impossible in gold filling and porcelain inlay work and, even were those walls strong enough to withstand the malleting of gold, the irregular line resulting would be very unsightly. A clean curved line although showing more gold is more pleasing. While with Silicate cements, on the contrary, the irregular line permits better blending of the filling material and the actual tooth structure.

I do not understand Dr. Ames to mean that he would allow any chalky or decalcified borders to remain. Altho this might be allowable in certain anterior teeth to preserve them for some time rather than to replace with crowns.

Many of us think we can put on pretty nice crowns, but unless the other teeth are of a very even type, our crowns will fail to have that home-like appearance which the original tooth had.

I see so many of those shell-like incisors in young mouths in the college clinic that I cannot always resist the temptation to put in an Ascher's when possible.

You may ask why, if I think this material often good enough for my private practice, I should hesitate to allow the students to use it? I hesitate because we are not yet quite sure how long a Silicate filling will last. I hesitate still more because, if it really is good, those who are to use it must help to make and keep it so by gaining the very best technique required for any of the fine work of our profession, and I believe there is nothing that gives so good a training as operating with gold.

Dr. Ames has referred in a very modest way to some Silicate cement of his own. He was good enough to send me samples of the same. I am sorry to say that I have not been able to use it for many practical cases as the time would not permit me to work out the colors, but I have this to say for it. When I did use it I thought it gave more time for inserting before it actually set, and what, to me, was very important, I was able to produce a very much better surface. It seems to set in much smaller crystals and, compared with Ascher's, it cuts and finishes with about the difference you find between cutting and finishing a Davis crown and a Logan crown. That is to say, when set, the Ames Berylite cement is of a much finer and closer texture and so takes on a polish that I, at least, have been unable to attain with the Ascher unless I was so fortunate as to have used just exactly the right amount, and as it is always easier to use an excess, so that there can be no doubt of the borders being full, this finishing quality of Ames Berylite is of exceeding importance.

In all cases the apparent excess of cement should be removed with sharp cutting instruments taking care to cut toward and over the enamel borders

I think one of my chief mistakes has been made in attempting to fill more than one cavity with a single mix. Another has been the using of too light a shade. If possible the color should always be determined before the dam has been placed, that is, before the teeth have dried out. In one or two cases, when the color did not prove just right, I have cut out the labial half of an incisor filling and replaced with a better shade. I find a small, sharp, round bur, the best for cutting out.

I have not felt quite sure about the liquid of the Ascher cement as some bottles have, on standing, a distinct white sediment while other bottles of the same lct and under the same conditions have no sediment. One seems to work as well as the other, but why this difference?

If Dr. Ames' Silica Jelly will remain constant in all weather it would seem that mcre definite and reliable results can be expected from that form of liquid than from one which requires shaking.

I have been much interested in that portion cf Dr. Ames' paper relating to the staining or darkening of Aschers' fillings. From time to time I have noticed a change in some fillings but I usually attributed this to faulty manipulation. I shall be glad now to make a systematic study to see if the cases in practice show the results Dr Ames has found in his experiments. I have a record of each filling with the colcrs used. Where more than cne color was used in the mix that combinaticn will have to be considered. Not being a chemist I shall leave the causes cf discoloration to be discussed by Dcctcrs Ward and Bunting. I think the reason abraided surfaces of silicate fillings show more discclcraticn than smooth surfaces do is that they present a greater surface.

As socn as I became thcroughly interested in Silicate cements I purchased a number cf tortcise shell and ivory instruments. I found them either too clumsy or so frail that they were soon broken either in their use or in careless cleaning. I am now using a number of various sized and shaped burnishers cast of gold and set in ordinary handles.

Before that I had a number of such instruments gold plated but I soon found the plating was being cut off by the abrasive cement. The instrument that I use most is a very thin but rigid steel burnisher that I use over celluloid for all labial or proximal surfaces. Upon occlusal surfaces I use the various sized round burnishers either in the hand or in the engine over celluloid.

I have much faith in Dr. Ames' method of hastening the crystallization with hot paraffine.

I saw this morning a patient in whose mouth I had recently placed two large incisor restorations. They were excessive restorations and were teeth which I should not care to risk filling with gold. I know very well I could have filled those cavities with gold with reasonable success knowing they would stay there for some time, but I don't know that porcelain inlays would stand in such cavities any better. I spent, I think, a couple of hours trying to produce a cement that would match up with these teeth, and it was fully two hours before I could get a combination that looked well in those teeth. I made a number of mixes and matched them with the teeth after they had set. The last one I put in. This is not the first time these teeth have been filled, by the way. I put these two enamel fillings in some time ago, and only one of them is broken out. I think it broke out because he had a nervous habit of rubbing his teeth together. I think he probably holds his pipe in the neighborhood of that tooth and wore it out in that way. Anyhow, I am comforting myself with that thought. I might say that I have been using a bone spatula. I have do doubt that they wear off some, but it does not seem to me enough of the bone would be incorporated into the surface of the filling to make any great amount of difference. The agate spatula may be best theoretically.

Dr. F. W. MacDonald.—I believe there is a tendency in the dental profession to-day to say a little too much of the Silicate cement; whether it is because we think we can get good results, or because it is a lot easier to manipulate, I do not know. The results that I have obtained in the use of Silicate cements have been so varied that I hardly know where to place it. So far I have used it in the small proximal cavities of the anterior teeth; outside of that, I have depended almost entirely on porcelain and the gold inlay.

Dr. Hall spoke about the technique necessary for the successful manipulation of the Silicate cement. I am thoroughly in accord with his ideas in the matter, and for that reason I think it was a splendid idea that we minimize its use among the students in college clinics. There is a tendency in manipulating any plastic for the operator who is not careful to be just a little bit careless and to perhaps not properly care for the finer details of manipulation. For that reason, I think it is a good idea for the new men in the profession to stay more closely by the older filling materials that have been tried and found good, and let some of the older men, who have made their reputations, stand the failures, if the Silicate should prove a failure. I know very little about the chemical aspect of Silicate cements. I do know that the color problem has been a very vexatious one with me. With some I get very good colors, and others I have spent so much time in matching colors and the results have been so poor that I have been sorry afterwards that I did not deal with porcelain or gold. I fancy that with all the time we spend on Silicate cements in endeavoring to get colors, and with the results not so sure, that perhaps it would be much better if we used the silicate cements simply in the smaller cavities, using them in that way because we do not need to extend the cavity margins as much as we should were we to fill with gold or porcelain. In that way, if the silicate cements do fail, and we do have to restore with porcelain or gold, the patient is not sacrificing very much.

Dr. M. L. Ward.—I have been very much impressed with Dr. Ames' modesty in not saying too much of his own product, and the remark he made about other dealers endeavoring to promote their products by too enthusiastic claims, that is one thing we cannot accuse him of. He has always given us information generously and been very modest about his own products. We should appreciate such professional conduct.

One thing I would like to speak of particularly in this connection, simply because it has impressed me a good deal, in the life as well as the looks of the silicate cements, and that is the retention of the surface which we get in the finishing. This is not only true with silicate cements, but it is true with almost all kinds of fillings that we do, the kind of surface has a great deal to do with the penetration of extraneous substances, and the surface color may be interfered with in a variety of ways. If you were to leave a filling of almost any description unfinished on its surface, it would be a very bad mechanical product. If we take any piece of metal that we are ready to polish and examine the exterior and interior before and after polishing we find that the surface is very much denser by the polishing. Take the hardest of our materials, such as the very hardest steel, and subject its surface to the polishing process, if then an examination is made with a microscope it will show the difference. It becomes as the engineers say, like metal that has been hammered. In some materials this is not true, some of the alloys of mercury are so soft that under a polishing wheel the surface cannot be made very much denser. I think that the surface of the filling should be retained intact and not ground down.

The action of the different sulphides on filling materials has occupied a good deal of attention for many years. Until recently the action of ammonium sulphide and likewise potassium and sodium sulphide has not been generally understood. The oxaphosphates have had their dissolving properties attributed to lactic acid, and the action of ammonia and ammonium salts has been overlooked. A good many have felt that there was not enough sodium, potassium, or ammonium in the mouth to account for the things often supposed to be due to soluble sulphides. There must be something, however, besides hydrogen sulphide and the only other conclusion to draw is that some one or more of the soluble sulphides are present in such cases. We can easily account for the presence of hydrogen sulphide but not quite so easily for the soluble sulphides. The question seems to be, "Is there sulphur?" We are certain of the ammonium and sodium in many cases but are not so certain about the sulphur. There is sulphur present, there is ammonium chloride and some sodium and potassium in many of the salivas, so that with this small percentage of sodium, potassium and ammonia we may get a little sodium sulphide, potassium sulphide and a little of ammonia sulphide, but it is not a question of whether they are there as such; it is a question of whether there is sulphur there in the most of these cases. Not only because there would be hydrogen sulphide, and in this case there will be acid reaction, but because sodium, potassium or ammonium would result in a soluble sulphide reacton and in this case there would be a basic reaction, "so that reactions are detected in both sides. I think ammonium sulphide is as liable to be present as sodium or potassium sulphide.

Discoloring of the teeth and filling material has been attributed to the sulphides, but the condition in which these sulphides exist has always been a question ,and until within a year I have not had the remotest idea that soluble sulphides discolored some metals worse than hydrogen sulphide does. I think he is justified in the test he has made showing it is from sulphide.

I use all steel instruments, because I could not get the others into some of the crevices in which I want to use them, I have been very careful about the rubbing. One part of the operation has been difficult to do without steel instruments, the finishing of proximal margins; if it slips over this margin and does not seem to rub it much, there is no discoloration. I use steel instruments in which I have taken white vaseline and rubbed over them and then rubbed off all I could with a cloth, and then rubbed with this, and have not seen any bad results. I have been forced to use it in some cases, because I could not use the agate instruments for want of room.

Dr. R. W. Bunting.—My experience with the silicate cements has been entirely negative. I have not learned to mix them so that they give me good results. I imagine that the nigger in the fence in my procedure has been brought out in Dr. Ames' paper to-night, and that is that I am using perhaps too much vaseline in my filling in my efforts to keep the instruments from affecting it.

Just one point that has occurred to me in this hydrogen sulphide discoloration. It seems to me that, as Dr. Ward says, we can have potassium sulphide which must come from the sulphides in the saliva or present in the mouth. The only forms in which I think sulphides can be present in the saliva is that of potassium sulphocyanides. Lately I have been making some experiments to ascertain the exact percentage of the potassium sulphocyanides in the various salivas and susceptible individuals in regard to caries, and I find that the percentage is extremely low, down on an average to one and two ten-thousandths of one per cent; the highest I have seen has been one exceptional case of a student in the college which gave a percentage of a two hundreths of one per cent. Now in view of this extremely low percentage of sulphides in the saliva, it seems difficult to say how very much hydrogen sulphide can be formed to cause any discoloration from the sulphide alone. If a hydrogen sulphide is formed, it seems that it must come as was suggested by Dr. Ames, from the putrefaction of extraneous materials, as may sometimes be seen in cases of caries in the occlusal surfaces of teeth where food gets crowded into the orifices of the cavities, putrefies and turns the whole surface of the cavity dark, probably from the hydrogen sulphide decomposition. These are present in some cases, and where there is much putrefaction in the mouth there might be enough hydrogen sulphide formed to cause considerable discoloration. It occurs to me that this hydrogen sulphide must be in mouths where there is uncleanliness, and in mouths where there is perfect hygiene there is little or no putrefaction of food and I do not see how there can be much hydrogen sulphide formed. I think in these mouths that the fillings ought to retain almost the normal color, or discolor very, very slowly.

DR. C. H. LAND.—I am very much interested indeed in this question as I have had a long experience in trying to make cements. There is one thing I would like to suggest, and that is that you soak your bone spatulas in hot paraffine for ten or fifteen minutes until they become thoroughly saturated, and then wipe them off, and you will get along better. Or take an orange wood spatula or one made of hard wood, and boil it in hot paraffine, there will be no trouble from the acid. Lactic acid has a great affinity for all kinds of metal, and unless the acid is neutral you are sure to have some trouble in a mix. I have had considerable experience with cements because of the many experiments I went through, and have little faith in them, so cannot say much in favor of the silicate cements.

DR. AMES.—The proper mix of Silicate cement is about the same as for oxaphosphate of zinc. There is a good deal of the personal element involved, a good deal in knowing how stiff it ought to feel under the spatula, and how the spatula will feel under his finger which is hard to describe, with these materials. I have been trying to learn what does give the best results, whether the quick mix, that is, the quick incorporation of powder with comparatively little spatulation, or a slow addition of powder with much spatulation. In adding powder quickly, or as fast as you can incorporate it until it is quite stiff, and then spatulating as much as that consistency will admit of before it will set is one method I have tried. Now in that there would be not a great deal of spatulation during the mix and a thorough spatulation after you felt you had in all the powder which ought to go in, and it would not be used quickly. The other method was adding powder slowly and ceasing adding powder at the point when the cement was becoming decidedly gummy, but not at a point at which you could not incorporate more powder if you wished. Now at that stage, with the long spatulation until you reach the actual tendency to set, it becomes quite gummy, I believe in this way I had a better mass. It seems to be harder and stronger and of a better transluscency.

In one plan I tried to describe incorporating all the powder that I could, adding it quickly, which gives a good chance to spatulate after I have put in all the powder I can. In that case it makes a coarser mass, and it does not seem to have quite as good an integrity and quite as nice a transluscency as if you cease adding powder when you might yet add some more. I would say cease adding the powder when it has yet plasticity but is decidedly gummy. After such a mix I have felt I observed a better texture and better transluscency and better properties generally. If I had been able to describe to you a process that has seemed best to me, that is the best I can do. I recently observed a representative of the Ascher enamel people in Chicago, who pretends to mix it according to the manufacturers methods, but I think he does not know the first principles, of mixing cements. He pats it a little and uses portions that do not properly mix.

At one time he said, don't spatulate it at all, or do not spatulate it during the mix, sort of "tease" it together, and when you have in ,say, $\frac{2}{3}$ enough powder spatulate it a little, a very little spatulation all told. I do not think he came anywhere near getting what is in the material, and he is supposed to be one of their apostles.

In regard to the life of a restoration, that brings up the question of the classes of cavities in which I believe these are fit to be used. I do not believe in large restorations at all, I believe they are to be used in small cavities which are found in their incipiency, which can be prepared by retaining much enamel, so that the matter of getting a fair shade is a small matter. I believe that in such cavities the life of an Ascher enamel filling, if a pigment in color is not used which will be black or brown in a little while, and the selection is where the sulphides are not formed from the food, the Ascher enamel will be good. I have seen some which have been in use a sufficient time, and which in a few years look as though they had been inserted last week, and look as if they might be lasting, but they may not.

I think there can be a range of conditions under which you might get complete crystallization if you applied heat for instance, but there will be a condition beyond that range where there would not be crystallization, and you would have free acid. You can set crowns with cement which is lasting and get thorough crystallization with it, you mix the cement stiff and so get thorough crystallization. There is a range in which you can get complete crystallization, there is also a point beyond that where you have mixed the cement so stiff that it is unmanageable. There also is a certain latitude you can go beyond and have free acid which will not be taken care of by the base. If you have a solution that is excessively acid it will leave you a honeycomb mass. There is little now I can say, except that I believe the permanency of these fillings depends upon the selection of cases. I believe the staining comes, in some cases, from the high percentage possibly of sulphocyandide of potassium, but more often from sulphureted hydrogen because of putrefaction.

DR. BOWLES.—I have been administering potassium sulphocyanide for some months, and in administering it there is a much more marked reaction in the mouth, so that the normal secretions is much less than ½ grain per day.

DR. AMES.—While I have felt that a study of the saliva would throw some light on this discoloration, I do not know anything of it except that I know that sulphuretted hydrogen can easily be present because of the putrefaction of the food. Now this matter of retaining the original surface is no doubt an important matter, but if the proper color of a cement filling depends upon the original surface being retained, that material is not a practical one to use, because we cannot put in a cement filling and expect that original surface to be present after a more or less short time, so that if the breaking or disappearance of the original surface is going to cause a bad result we had better cure that by not beginning. I am inclined to believe that steel instruments have been blamed for much discoloration which has really depended upon some inherent defect in the material, such as improper pigment. What that pigment is, I have not been able to ascertain. I do happen to know that cobalt blue does not give serious stain, that platinum black gives a grey which will not be affected by these pigments, and what is used in these cements which will not change the colors more than the others I do not know. Possibly if the makers of these cements will get busy and produce different pigments they may produce a much more valuable material.

